# The essential *M. tuberculosis* Clp protease is functionally asymmetric in vivo

**DOI:** 10.1126/sciadv.abn7943

**Published:** 2022-05-04

**Authors:** Felipe B. d’Andrea, Nicholas C. Poulton, Ruby Froom, Kayan Tam, Elizabeth A. Campbell, Jeremy M. Rock

**Affiliations:** 1 Laboratory of Host-Pathogen Biology, The Rockefeller University, New York, NY, USA.; 2 Weill Cornell/Rockefeller/Sloan Kettering Tri-Institutional MD-PhD Program, Weill Cornell Medicine, New York, NY, USA.; 3 Laboratory of Molecular Biophysics, The Rockefeller University, New York, NY, USA.

## Abstract

The Clp protease system is a promising, noncanonical drug target against *Mycobacterium tuberculosis* (Mtb). Unlike in *Escherichia coli*, the Mtb Clp protease consists of two distinct proteolytic subunits, ClpP1 and ClpP2, which hydrolyze substrates delivered by the chaperones ClpX and ClpC1. While biochemical approaches uncovered unique aspects of Mtb Clp enzymology, its essentiality complicates in vivo studies. To address this gap, we leveraged new genetic tools to mechanistically interrogate the in vivo essentiality of the Mtb Clp protease. While validating some aspects of the biochemical model, we unexpectedly found that only the proteolytic activity of ClpP1, but not of ClpP2, is essential for substrate degradation and Mtb growth and infection. Our observations not only support a revised model of Mtb Clp biology, where ClpP2 scaffolds chaperone binding while ClpP1 provides the essential proteolytic activity of the complex; they also have important implications for the ongoing development of inhibitors toward this emerging therapeutic target.

## Teaser

Functional asymmetry: Mtb ClpP2 engages chaperones, while ClpP1 provides the essential proteolytic activity of the Clp complex.

## INTRODUCTION

Ubiquitous in its role as a regulator of pathogen physiology and virulence, the caseinolytic protease (Clp) system has emerged as a high-priority antimicrobial target (*1*–*3*). In contrast to other bacteria, the proteolytic and chaperone components of the Clp system in *Mycobacterium tuberculosis* (Mtb), the causative agent of tuberculosis, are essential and genetically vulnerable (*4*). The Clp system in mycobacteria is structurally asymmetric and is composed of a two-tiered proteolytic barrel of homoheptameric ClpP1 and ClpP2 rings that degrades substrates delivered by the AAA+ chaperones, ClpX and ClpC1 (*5*). In the absence of chaperone binding, the axial pores of the ClpP1P2 proteolytic chamber are closed, and its catalytic triads are disordered to prevent nonspecific proteolysis (*6*–*8*). The current model of the mycobacterial Clp system asserts that chaperones deliver substrates by docking to hydrophobic clefts on the ClpP2 surface. In vitro studies using proteolytically inactivated subunits demonstrated that ClpP1 exhibited much greater activity toward small peptides than ClpP2. However, both protease subunits were capable of comparable rates of model protein degradation, suggesting proteolytic redundancy in the Mtb ClpP1P2 complex (*6*, *9*, *10*). Despite this apparent redundancy in vitro, both ClpP1 and ClpP2 proteolytic activity are proposed to be necessary for Mtb growth and pathogenesis (*9*, *11*, *12*). Because of the historical technical challenges posed by studying essential Clp genes in mycobacteria, correlations between biochemical studies and in vivo function remain scarce (*12*–*14*). As a biologically attractive (*4*, *12*, *13*) and chemically validated (*2*, *10*, *15*–*19*) noncanonical drug target, a deeper mechanistic understanding of the ClpP1P2 complex in vivo is critical to guiding ongoing drug discovery efforts and further our understanding of Clp biology in Mtb pathogenesis.

## RESULTS AND DISCUSSION

### ClpP2 protease activity is nonessential for Mtb growth in laboratory culture

**Fig. 1.**
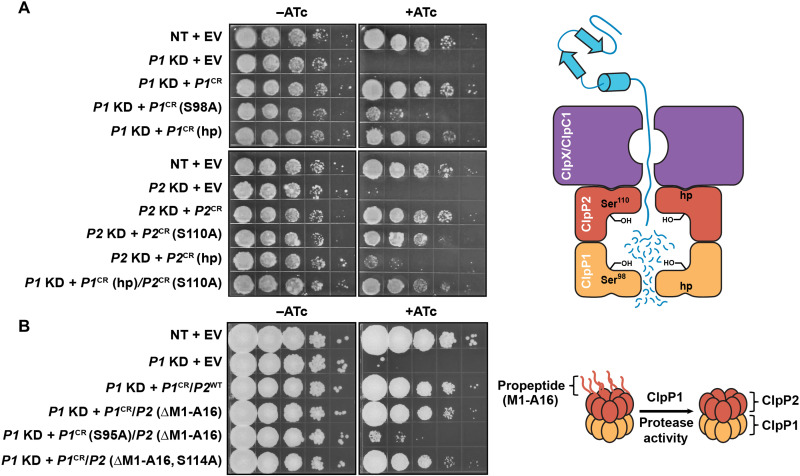
The Mtb Clp protease is functionally asymmetric in vivo. (**A**) Growth of the indicated Mtb CRISPRi strains. ATc induces expression of dCas9 and the single-guide RNA (sgRNA). NT, nontargeting sgRNA; EV, empty complementation vector; KD, knockdown; S98, Mtb ClpP1 catalytic serine; CR, CRISPRi-resistant allele; S110, Mtb ClpP2 catalytic serine; hp, hydrophobic patch mutations: P1, S61A, Y63V, L83A, and Y91V; P2, Y75V and Y95V. (**B**) Growth of the indicated Msmeg CRISPRi strains. S95, Msmeg ClpP1 catalytic serine; S114, Msmeg ClpP2 catalytic serine; ΔM1-A16, Msmeg *clpP2* allele expressed without the propeptide; WT, wild type.

To investigate the molecular roles of each Clp proteolytic subunit in vivo, a robust, inducible CRISPR interference (CRISPRi) approach was undertaken in both Mtb and the model mycobacterium *Mycobacterium smegmatis* (Msmeg) (*20*). Briefly, phenotypically strong knockdowns of endogenously encoded *clp* genes were complemented by CRISPRi-resistant *clp* alleles harboring desired functional mutations (*21*). Complementation constructs were expressed from native promoters to minimize expression-level artifacts. Mutations were selected on the basis of residues biochemically validated for functional activity (*9*, *11*, *12*). As expected, knockdown of *clpP1* or *clpP2* by addition of the CRISPRi inducer anhydrotetracycline (ATc) prevented growth in both Mtb and Msmeg (Fig. 1A and fig. S1, A and B) (*12*). Growth was restored by complementation with CRISPRi-resistant alleles, demonstrating specificity for the observed phenotypes. Because of the *clpP* operon structure, *clpP1* knockdown strains were complemented with the full *clpP1P2* operon, whereas *clpP2* knockdown strains were complemented with *clpP2* alone. Mutation of the ClpP hydrophobic patches predicted to be necessary for interaction with the ClpX and ClpC1 AAA+ ATPase chaperones blocked growth when mutated in ClpP2 but not in ClpP1, consistent with the accepted Clp model derived from biochemical and inhibitor-binding observations that ClpP2 exclusively serves as the docking site for these essential chaperones (Fig. 1A and fig. S1, A and B) (*7*, *9*, *11*). On the basis of previous investigations using inducible depletion systems, the proteolytic activity of both ClpP1 and ClpP2 subunits was expected to be essential (*12*, *14*). Consistent with these prior reports, inactivation of ClpP1 protease activity by mutation of the catalytic serine to alanine (S98A) prevented mycobacterial growth (Fig. 1A and fig. S1, A and B). The *clpP1* (S98A) and *clpP2* hydrophobic-patch mutant alleles expressed at similar or higher levels than wild-type *clpP1P2* (fig. S1C), consistent with biochemical observations that mutating these residues does not disrupt protein folding (*9*, *11*). The increased expression of the mutant alleles in the presence of ATc is likely due to up-regulation of the *clgR* regulon as a result of inactivation of the ClpP1P2 protease (*22*, *23*). In contrast to ClpP1, however, inactivation of ClpP2 proteolytic activity unexpectedly had little to no effect on mycobacterial growth (Fig. 1A and fig. S1, A and B), suggesting that ClpP2 protease activity was not essential under these laboratory culture conditions. Together, these results suggest that the Mtb Clp protease is functionally asymmetric in vivo, with ClpP2 providing the essential ClpC1 and ClpX docking surface and ClpP1 providing the essential proteolytic activity of the complex. Consistent with this model, a “forced asymmetry” strain, in which the ClpP1 hydrophobic patch and ClpP2 proteolytic activity were simultaneously mutated, grew similarly to control strains (Fig. 1A and fig. S1, A and B).

One potential explanation for the essentiality of ClpP1 proteolytic activity could be its unique role in assembling the mature ClpP1P2 complex. ClpP2 in Msmeg and both ClpP1 and ClpP2 in Mtb are expressed with N-terminal propeptides, which are proteolytically removed by ClpP1 during complex maturation (*9*, *11*). To test this hypothesis, we complemented *clpP1P2* knockdown with matured (i.e., propeptide-truncated) *clpP1P2* alleles. Propeptide-truncated alleles rescued growth of *clpP1P2* knockdown (Fig. 1B), demonstrating that the propeptide-truncated ClpP1P2 complex is functional. However, the lack of ClpP1 proteolytic activity prevented growth even in the context of propeptide truncation (Fig. 1B), suggesting that ClpP1 proteolytic essentiality cannot be simply attributed to its role in ClpP subunit maturation.

### ClpP1 proteolytic activity is necessary for the turnover of Clp substrates

**Fig. 2.**
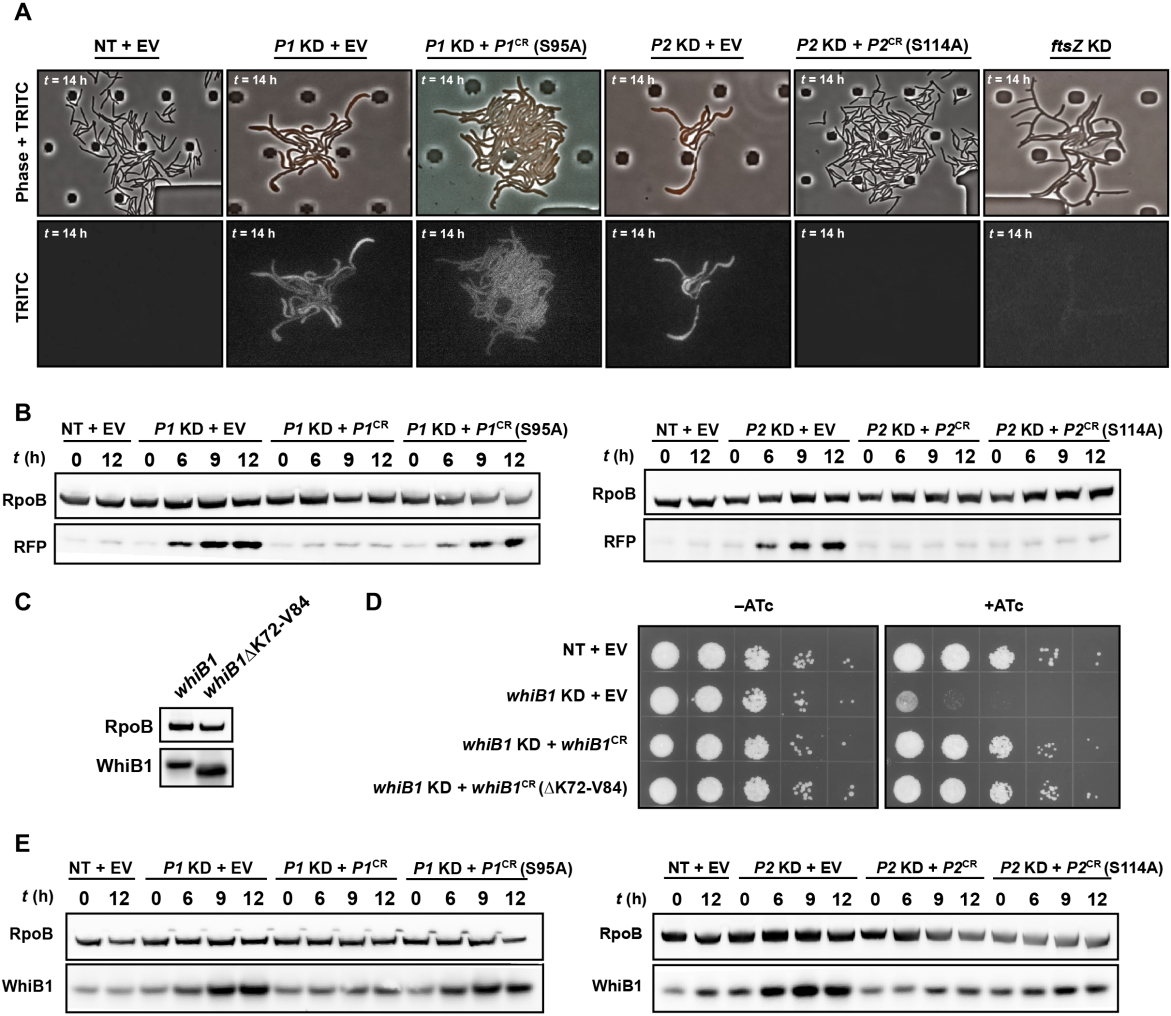
ClpP1, but not ClpP2, proteolytic activity is necessary and sufficient for the turnover of mScarlet-YALAA and WhiB1. (**A** and **B**) Time-lapse microscopy (A) and immunoblot (B) measuring mScarlet-YALAA accumulation in the indicated Msmeg CRISPRi strains. *t* (h), hours after ATc addition. (**C**) Immunoblot of Msmeg 3xFLAG-WhiB1 and C-terminally truncated Msmeg 3xFLAG-WhiB1ΔK72-V84 from log-phase bacterial cultures. (**D**) Growth of the indicated Msmeg CRISPRi strains. ΔK72-V84, WhiB1 C-terminal truncation. (**E**) Immunoblot measuring 3xFLAG-WhiB1 levels in the indicated Msmeg CRISPRi strains. TRITC, tetramethyl rhodamine isothiocyanate.

An alternative explanation for the essentiality of ClpP1 proteolytic activity is that ClpP1 is uniquely essential for the degradation of particular Clp substrates in vivo. While consistent with the observed bacterial growth phenotypes in vivo, this explanation would be unexpected given the apparent biochemical redundancy of ClpP1 and ClpP2 proteolytic activity against model protein substrates in vitro (*9*, *10*). To test this hypothesis, we monitored known Clp substrate levels in the presence or absence of ClpP1 or ClpP2 protease activity. We first appended the C-terminal five amino acids of the Msmeg ssrA degradation tag (YALAA), a known Clp degron that promotes turnover of trans-translation products (*24*), to the fluorescent protein mScarlet. As expected, depletion of both ClpP1 and ClpP2 subunits resulted in mScarlet-YALAA accumulation (Fig. 2, A and B). Depletion of ClpP1P2 led to severe growth and division defects, with individual cell poles bulging and lysing (S.V. 1-8) (*25*). Consistent with the hypothesis that ClpP1 protease activity is uniquely essential for substrate degradation, mScarlet-YALAA accumulated in the absence of ClpP1 but not ClpP2 protease activity (Fig. 2, A and B). mScarlet-YALAA accumulation was independent of changes in target mRNA levels (fig. S2, A and B). Knockdown of another essential gene, *ftsZ*, produced similar growth inhibition and morphological defects but did not lead to mScarlet-YALAA accumulation.

We next sought to validate these observations with the endogenous Clp substrate, WhiB1. WhiB1 encodes a C-terminal degron necessary and sufficient for Clp-mediated turnover (*13*). Deletion of the WhiB1 C-terminal degron led to WhiB1 accumulation as expected Fig. 2C, but contrary to a previous report (*13*), this accumulation was not lethal, arguing against an essential role for the Clp protease in mediating WhiB1 turnover (Fig. 2D). These results are consistent with recent site-directed mutagenesis studies, which showed that truncating the WhiB1 C terminus in Msmeg did not affect growth (*26*). We next assessed WhiB1 accumulation in the absence of Clp activity. Depletion of ClpC1, but not ClpX, led to WhiB1 accumulation, consistent with ClpC1-dependent delivery to ClpP1P2 (fig. S2, C and D). As with mScarlet-YALAA, ClpP1 but not ClpP2 protease activity was essential to prevent WhiB1 accumulation (Fig. 2E). As with mScarlet-YALAA, WhiB1 accumulation in the absence of ClpP1 protease activity was not associated with increases in *whiB1* mRNA levels (fig. S2, A and B), demonstrating that WhiB1 accumulation occurred posttranscriptionally. Together, our results show that, although ClpP2 is essential for the delivery of substrates to the proteolytic complex, ClpP1 proteolytic activity alone is necessary and sufficient for degradation of at least some Clp substrates and provides the essential proteolytic function of the Mtb ClpP1P2 protease under these growth conditions.

### ClpP2 proteolytic activity is nonessential during acute and chronic infection in mice

**Fig. 3.**
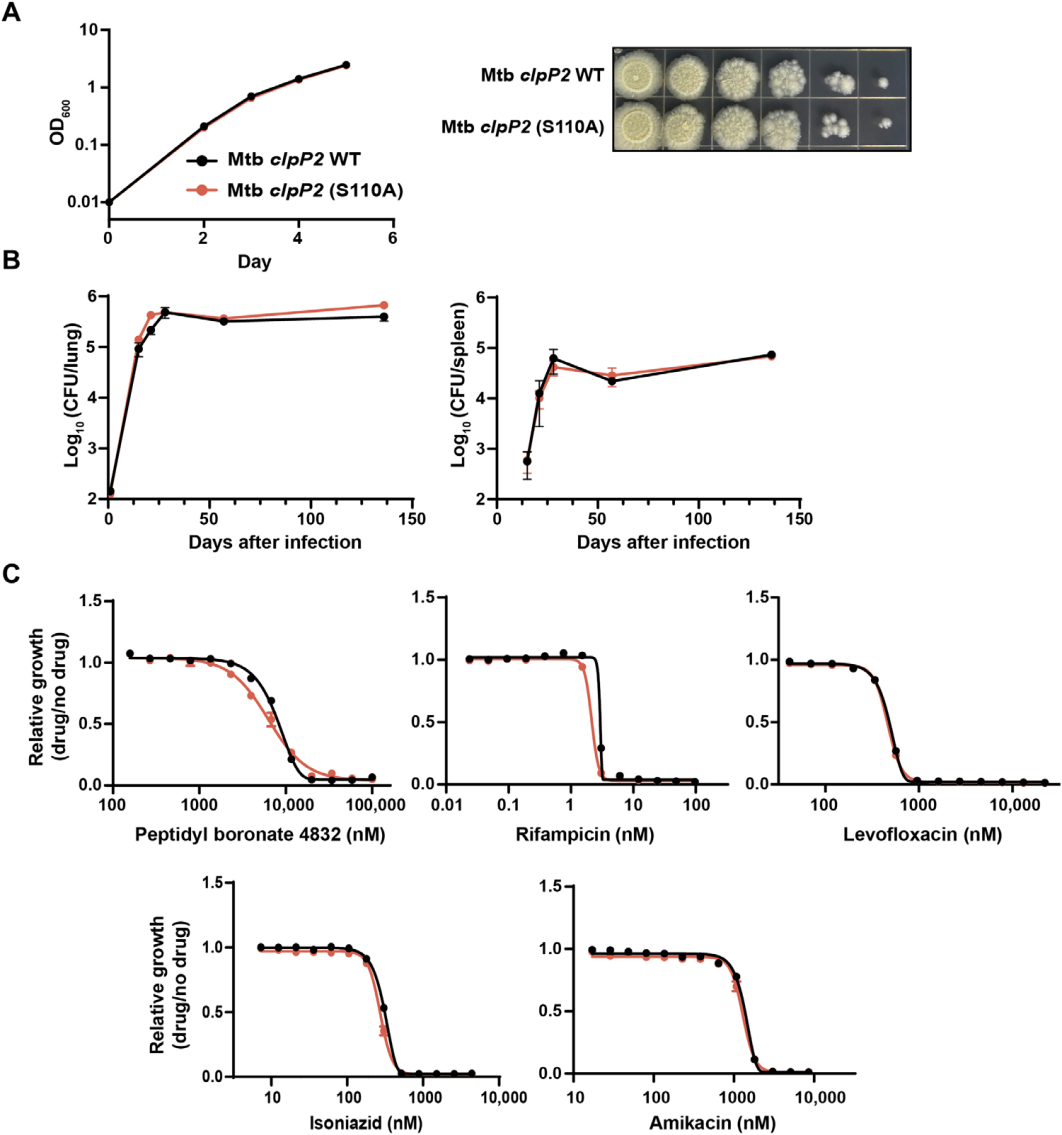
Mtb ClpP2 proteolytic activity is nonessential during mouse infection or antibiotic treatment. (**A**) Growth of the indicated recombineered Mtb strains. Data represent the mean of eight technical triplicates ± SD. OD_600_, optical density at 600 nm. (**B**) Colony-forming units (CFU) in lungs and spleens of C57BL/6J mice low-dose aerosol–infected with Mtb strains shown in (A). Data are mean CFUs per organ ± SD of five mice. (**C**) Minimum inhibitory concentration curves of recombineered Mtb strains with the indicated antibiotics. Data represent the mean of technical triplicates ± SD.

Recently, several classes of natural products and synthetic small molecules have been found to disrupt the mycobacterial Clp system (*2*). Given the implications of our results for ongoing efforts to develop ClpP1P2 protease inhibitors as new antituberculars, we next sought to determine whether ClpP2 protease activity may be essential in the context of infection. During infection, Mtb bacilli are exposed to a broad range of noxious host stimuli including reactive oxygen/nitrogen species and nutrient starvation (*27*) that could strain the Mtb proteostasis network and potentially reveal an essential role for ClpP2 protease activity. To perform these experiments, we first generated a proteolytically dead ClpP2 Mtb strain by recombineering (*28*). Successful mutation of the endogenous *clpP2* catalytic serine (S110A) and lack of any off-target or compensatory mutations were confirmed by whole-genome sequencing. Consistent with our prior results, the proteolytically dead *clpP2* (S110A) Mtb strain grew with near-identical kinetics to the control strain under laboratory culture conditions (Fig. 3A). To test the hypothesis that ClpP2 proteolytic activity may be essential during host infection, we performed a low-dose aerosol infection of C57BL/6J mice. No substantial differences in lung and spleen bacterial burdens or histopathology were observed between mice infected with the *clpP2* (S110A) recombineered and control Mtb strains, indicating that ClpP2 protease activity is nonessential for virulence during acute and chronic infection in this mouse model (Fig. 3B and fig. S3).

The peptidyl boronates, a class of electrophilic serine protease inhibitors, have shown promising whole-cell tuberculocidal activity through inhibition of ClpP1P2 protease activity (*10*, *15*). Our results demonstrate that ClpP2 protease activity is nonessential under the conditions assayed. Thus, we hypothesized that peptidyl boronates and similar protease active site–targeting compounds exert their antibacterial activity by inhibiting ClpP1 and not ClpP2. Consistent with this prediction, we found that the *clpP2* (S110A) recombineered strain was not more sensitive than the control strain to peptidyl boronates or other antibiotic classes (Fig. 3C). These results strongly support a model whereby ClpP1 furnishes the essential proteolytic activity of the complex and should be prioritized for inhibition over ClpP2 by drug discovery campaigns.

While structural and biochemical studies laid much of the foundation for our current knowledge of the mycobacterial Clp system, in vivo mechanistic understanding is critical to advancing ClpP1P2 as a drug target. By leveraging new and broadly applicable genetic approaches, we investigated the essential contributions of the ClpP1 and ClpP2 subunits to the mycobacterial Clp system. Our unexpected observations lead us to propose a revised model of the Clp system in mycobacteria: ClpP2 essentiality stems from serving as a scaffold for AAA+ chaperone binding and substrate delivery (*9*, *11*), not from its active participation in proteolysis. Conversely, ClpP1 exclusively furnishes the essential proteolytic activity of the complex, at least under the conditions tested. To explore whether functionally asymmetric Clp complexes may occur in other bacterial species, we examined conservation of the Ser-His-Asp catalytic triad residues in *clpP* homologs of diverse bacterial species. This analysis revealed widespread conservation of Clp protease active site residues with some exceptions (fig. S4). For example, *Nocardia asteroides*, *Streptomyces coelicolor*, and *Leptospira interrogans* encode multiple *clpP* alleles, with one allele lacking residues predicted to be critical for protease activity. This is reminiscent of the situation found in photosynthetic organisms such as *Synechococcus* spp. and *Arabidopsis* spp., which encode proteolytically inactive ClpR subunits whose function remains poorly understood (*29*, *30*). Given that other features of the Mtb Clp protease, for example, asymmetric binding of chaperones to one surface of the complex, are shared between Mtb and other multi-*clpP* allele–encoding bacteria (*31*–*34*), it is possible that functional asymmetry in Clp proteolysis may exist in other bacterial species as well.

The fact that Mtb ClpP1 proteolytic activity is essential while ClpP2 activity is not could be a result of their different substrate-binding pocket preferences (*7*, *10*) and/or cleavage kinetics between ClpP1 and ClpP2. For example, ClpP1 protease activity could be critical to producing a growth-promoting peptide or cleaving a growth-inhibitory peptide, analogous to the proposed role of ClpP2 in *Pseudomonas* biofilm formation (*33*). However, we favor a model whereby ClpP1 protease activity is critical to maintaining flux through the Clp system. In the absence of ClpP1 protease activity, the ClpP1P2 complex becomes clogged and inactivated. This latter model is consistent with the accumulation of mScarlet-YALAA and WhiB1 substrates in the absence of ClpP1 proteolytic activity, an observation difficult to reconcile with the growth-trophic peptide model. That truncation of the C-terminal degron leads to nontoxic accumulation of WhiB1 argues against a WhiB1-dependent role for Clp essentiality (*13*), suggesting that the dysregulation of another substrate underlies the essential function of the Clp system. Last, our results suggest that the ongoing pursuit of small-molecule therapeutics aimed at inhibiting ClpP1P2 protease activity should account for its functional asymmetry and prioritize the inhibition of essential ClpP1 subunit proteolytic activity.

## MATERIALS AND METHODS

### Bacterial strains

Mtb strains were derived from H37Rv, and Msmeg strains were derived from mc^2^155.

### Mycobacterial cultures

Mtb and Msmeg were cultured at 37°C in Difco Middlebrook 7H9 broth (BD, #271310) or 7H10 (BD, #262710) plates supplemented with 0.2% glycerol (7H9) or 0.5% glycerol (7H10), 0.05% Tween 80, and 1× albumin dextrose catalase (ADC; Msmeg) or oleic acid ADC (OADC; Mtb). Where required, antibacterials or small molecules were used at the following concentrations: ATc (100 ng ml^−1^), kanamycin (KAN; 20 μg ml^−1^), nourseothricin (25 μg ml^−1^), and zeocin (20 μg ml^−1^). Mtb cultures were grown standing in tissue culture flasks (unless otherwise indicated) at 37°C and 5% CO_2_. Msmeg cultures were grown shaking in flasks at 37°C and 120 rpm.

### Preparation of individual CRISPRi and CRISPRi-resistant complementation strains

plRL58 (Addgene, #166886) and plRL61 (Addgene, #163633) were used to generate all Mtb and Msmeg CRISPRi strains, as previously described by Rock and colleagues (*4*, *21*). plRL58 and plRL61 integration into the mycobacterial chromosome is mediated by the L5 integrase, supplied in trans on a separate nonreplicating suicide vector, plRL19 (Addgene, #163634). The CRISPRi plasmid backbone was digested using BsmBI–v2 (New England Biolabs, #R0739L) and agarose gel–purified. Single-guide RNAs (sgRNAs) bearing appropriate overhangs and designed to hybridize to the nontemplate strand of target gene open reading frames were annealed and ligated (T4 ligase, New England Biolabs, #M0202M) into the digested backbone. Identification of desired clones was confirmed by Sanger sequencing.

Individual CRISPRi strains were prepared by electroporation of plasmids into Mtb or Msmeg (*21*, *35*). For Mtb strains, a 20-ml culture of H37Rv was expanded to an OD_600_ (optical density at 600 nm) of 0.8 to 1.0 and pelleted (4000*g*, 10 min) before being washed with 10% glycerol (3 × 20 ml) and resuspended in 1 ml of 10% glycerol. For each transformation, 100 μl of electrocompetent cells was combined with ~100 ng of CRISPRi plasmid DNA and plRL19 before electroporation in a 2-mm cuvette (Bio-Rad, #1652082) using the Gene Pulser X cell electroporation system (Bio-Rad, #1652660) set at 2500 V, 700 ohms, and 25 μF. Electroporated cells were recovered in 7H9 broth overnight before being plated on 7H10 agar supplemented with KAN (20 μg/ml) for 18 to 21 days to select for transformants. Msmeg electrocompetent cells were similarly prepared and electroporated, except recovery in 7H9 broth occurred over 3 hours, and transformants were selected after 3 days.

CRISPRi-resistant complementation constructs were generated in Tweety-integrating (plRL91) or Giles-integrating (plRL125) backbones. Briefly, CRISPRi-resistant complementation alleles were amplified from Mtb or Msmeg genomic DNA using Gibson assembly primers encoding synonymous mutations in the complementation allele protospacer adjacent motif and sgRNA seed sequence to prevent sgRNA targeting (*21*). The amplified, CRISPRi-resistant allele fragments were then cloned into HF–Dra III (New England Biolabs, #R3510S)–digested plRL91 or HpaI (New England Biolabs, #R0105S)–digested plRL125 backbones by Gibson assembly (New England Biolabs, #E2621S). CRISPRi-resistant complement alleles were expressed under the control of their endogenous promoters. plRL91-derived constructs were cotransformed into mycobacteria with plRL62, a nonreplicating vector supplying the Tweety integrase in trans. plRL125-derived constructs were cotransformed with plRL40, a nonreplicating vector supplying the Giles integrase.

### Preparation of the Mtb *clpP2* (S110A) strain by single-stranded DNA recombineering

The Mtb *clpP2* (S110A) strain was constructed using a RecT-promoted, oligo-mediated recombineering approach as previously reported (*36*). To select for recombineered mutants, a RecT-expressing strain was cotransformed with an oligonucleotide conferring streptomycin resistance (*rpsL* K43R) and an oligonucleotide encoding the *clpP2* (S110A) mutation. Following transformation and recovery, cells were screened on 7H10 plates with streptomycin at 20 μg ml^−1^. A recombineered control strain with only the *rpsL* (K43R) mutation was similarly prepared. Selective mutation of the *clpP2* locus was confirmed by Sanger and whole-genome sequencing [Beijing Genomics Institute (BGI)]. No additional mutations were found between recombineered strains aside from the *clpP2* (S110A) point mutation. Oligonucleotide sequences can be found in table S1.

### Growth assays

To quantify growth phenotypes on 7H10 agar, 10-fold serial dilutions of Mtb or Msmeg cultures (OD_600_ of 0.6) were spotted on 7H10 agar with ATc at 100 ng ml^−1^. Plates were incubated at 37°C and imaged after 2 to 3 weeks (Mtb) or 3 days (Msmeg). Liquid growth assays were performed in 384-well plates (Greiner, #781091). Mtb cultures were growth-synchronized and then back-diluted to a starting OD_600_ of 0.01 before 50 μl of cell suspension was plated in the presence of ATc at 100 ng ml^−1^ with eight technical replicates per strain. Plates were incubated at 37°C with 5% CO_2_. OD_600_ was evaluated using a Tecan Spark plate reader.

### Time-lapse microscopy

Imaging was carried out using an inverted Nikon ECLIPSE Ti-2 microscope at ×60 magnification. Knockdown/complementation strains harboring a constitutively expressing mScarlet-YALAA construct were grown under constant flow of 7H9 supplemented with ATc at 100 ng ml^−1^ in a CellASIC ONIX2 Microfluidic System (MilliporeSigma, #CAX2-S0000, #B04A-03-5PK) placed in a 37°C environmental chamber. Phase and fluorescence channels (Chroma, #49005; excitation, 545 nm; emission, 620 nm) were imaged every 15 min. Image processing was performed using NIS Elements AR (Nikon) and Fiji (https://imagej.net/software/fiji/).

### Immunoblotting

Msmeg cultures were growth-synchronized before ATc was added to a final concentration of 100 ng ml^−1^. For each time point, 20 OD_600_ units of culture were harvested by centrifugation (4000*g*, 10 min) and resuspended in 600 μl of lysis buffer [50 mM tris and 150 mM NaCl (pH 7.4)] containing a protease inhibitor cocktail (Sigma-Aldrich, #11873580001) before lysis by bead beating in Lysis B Matrix tubes (MP Biomedicals, #116911050) using a Precellys Evolution homogenizer (Bertin Instruments, #P000062-PEVO0-A; 3 × 10,000 rpm, 30-s intervals). The cell lysates were cleared by centrifugation (20,000*g*, 10 min), and a 20-μl aliquot was incubated with 4× lithium dodecyl sulfate (LDS) sample buffer (Invitrogen, #NP0007) supplemented with dithiothreitol at 70°C for 10 min. Samples were separated on a 4 to 12% bis-tris polyacrylamide gel (Invitrogen, #NP0323BOX) in Mops or MES running buffer, transferred to a nitrocellulose membrane using the TransBlot Turbo Transfer System (Bio-Rad, #1704150), and incubated for 1 hour in blocking buffer (LI-COR, #927-60001). Proteins were probed with anti-RpoB (BioLegend, #663905), anti–red fluorescent protein (RFP; Rockland, #600-401-379), or anti-FLAG (Sigma-Aldrich, #F3165) primary antibodies overnight at 4°C and subsequently detected with fluorescent goat anti-mouse and anti-rabbit secondary antibodies (Bio-Rad, #12004159 and #12004162).

### Quantitative reverse transcription polymerase chain reaction

mRNA extraction was performed as previously described (*20*). Briefly, 2 OD_600_ units of Msmeg culture were directly pelleted by centrifugation (4000*g*, 10 min), resuspended in 1 ml of TRIzol (Thermo Fisher Scientific, #15596026), and lysed by bead beating in Lysis B Matrix tubes (MP Biomedicals, #116911050) using a Precellys Evolution homogenizer (Bertin Instruments, #P000062-PEVO0-A; 3 × 10,000 rpm, 30-s intervals). Chloroform (0.2 ml) was added to each sample, samples were centrifuged (15 min, 13,000 rpm) to separate phases, and the aqueous phase was purified by Direct-zol RNA miniprep (Zymo Research, #R2052). Residual genomic DNA was removed by TURBO DNase treatment (Invitrogen Ambion, #AM2238). After RNA cleanup and concentration (Zymo Research, #R1017), 3 μg of RNA per sample was reverse-transcribed into complementary DNA (cDNA) using random hexamers (Thermo Fisher Scientific, #18-091-050). RNA was removed by alkaline hydrolysis, and cDNA was purified with QIAGEN polymerase chain reaction (PCR) clean-up columns (#28106). Expression of genes of interest was quantified by SYBR green dye–based quantitative real-time PCR (Applied Biosystems, #4309155) on a Quantstudio system 5 (Thermo Fisher Scientific, #A28140) using gene-specific quantitative PCR (qPCR) primers (2.5 μM). Expression levels were normalized to *sigA* (*ms2758*) and quantified by the ΔΔCt algorithm. Relative fold change was determined relative to the 0-hour condition for each strain. All gene-specific qPCR primers were designed using the PrimerQuest tool from IDT (www.idtdna.com/pages/tools/primerquest?returnurl=%2FPrimerQuest%2FHome%2FIndex) and then validated for efficiency and linear range of amplification using standard qPCR approaches. Specificity was confirmed for each validated qPCR primer pair by melt curve analysis.

### Mouse aerosol infection

Sixty 7- to 8-week-old female C57BL/6J mice (Jackson Laboratory) were infected using an inhalation exposure system (Glas-Col) with OD_600_ 0.2 cultures of recombineered Mtb strains delivering approximately 100 to 200 bacilli per lung 1 day after infection. At each time point, lungs and spleens from five mice were homogenized in phosphate-buffered saline using a gentleMACS Octo Dissociator (Miltenyi Biotec, #130095937) using the RNA_01 program before bacteria were enumerated by plating serially diluted homogenates on 7H10 agar. Mice were housed in groups of five in individually ventilated cages inside a certified ABSL-3 (animal biosafety level 3) facility and had access to water and food ad libitum for the duration of the study. All experiments involving animals were approved by the Institutional Animal Care and Use Committee of The Rockefeller University.

### Antibacterial activity measurements

All compounds were dissolved in dimethyl sulfoxide (DMSO; VWR #V0231) and dispensed using an HP D300e Digital Dispenser in a 384-well plate format (Greiner, #781091). DMSO did not exceed 1% of the final culture volume and was maintained at the same concentration across all samples. Cultures were growth-synchronized and then back-diluted to a starting OD_600_ of 0.01, and 50 μl of cell suspension was plated in technical triplicate in wells containing compound. Plates were incubated at 37°C with 5% CO_2_. OD_600_ was evaluated using a Tecan Spark plate reader at 7 to 10 days after plating, and percent growth was calculated relative to the vehicle control for each strain. Dose-response curves were plotted using a nonlinear fit in GraphPad Prism.

### Phylogenetic analysis

Bacterial protein sequences were downloaded from the National Center for Biotechnology Information using the “eutils” online application programming interface. ClpP homologs in the modified, curated bacterial species set from (*37*) were identified by performing protein sequence searches with BLAST against a protein database containing Mtb ClpP1 and ClpP2. BLAST hits were required to have an *e* value of <1 × 10^−5^ and an identity of >35% to filter out spurious matches (*38*). ClustalW was used to produce both the multiple sequence alignment and the phylogenetic tree of the resulting ClpP homologs using the “Slow/Accurate” and “Tree based iteration” settings (*39*). Tree analysis and visualization were carried out with iTOL (*40*).

## Notes

View/request a protocol for this paper from *Bio-protocol*.
