# Supplementary material for: The essential M. tuberculosis Clp protease is functionally asymmetric in vivo
Source: Sci Adv. 2022 May 4;8(18):eabn7943. doi: 10.1126/sciadv.abn7943 (PMC9067928; doi:10.1126/sciadv.abn7943)
Supplement: Supplementary file 1 — Figs. S1 to S4 Table S1 [file sciadv.abn7943_sm.pdf]

Science Advances
AAAS

Supplementary Materials for

The essential *M. tuberculosis* Clp protease is functionally asymmetric in vivo

Felipe B. d'Andrea, Nicholas C. Poulton, Ruby Froom, Kayan Tam,
Elizabeth A. Campbell, Jeremy M. Rock*

*Corresponding author. Email: rock@rockefeller.edu

Published 4 May 2022, *Sci. Adv.* **8**, eabn7943 (2022)
DOI: 10.1126/sciadv.abn7943

The PDF file includes:

Figs. S1 to S4
Table S1
Legends for movies S1 to S8
Legends for data S1 to S3

Other Supplementary Material for this manuscript includes the following:

Movies S1 to S8
Data S1 to S3

SUPPLEMENTAL INFORMATION

Supplemental Figures

A

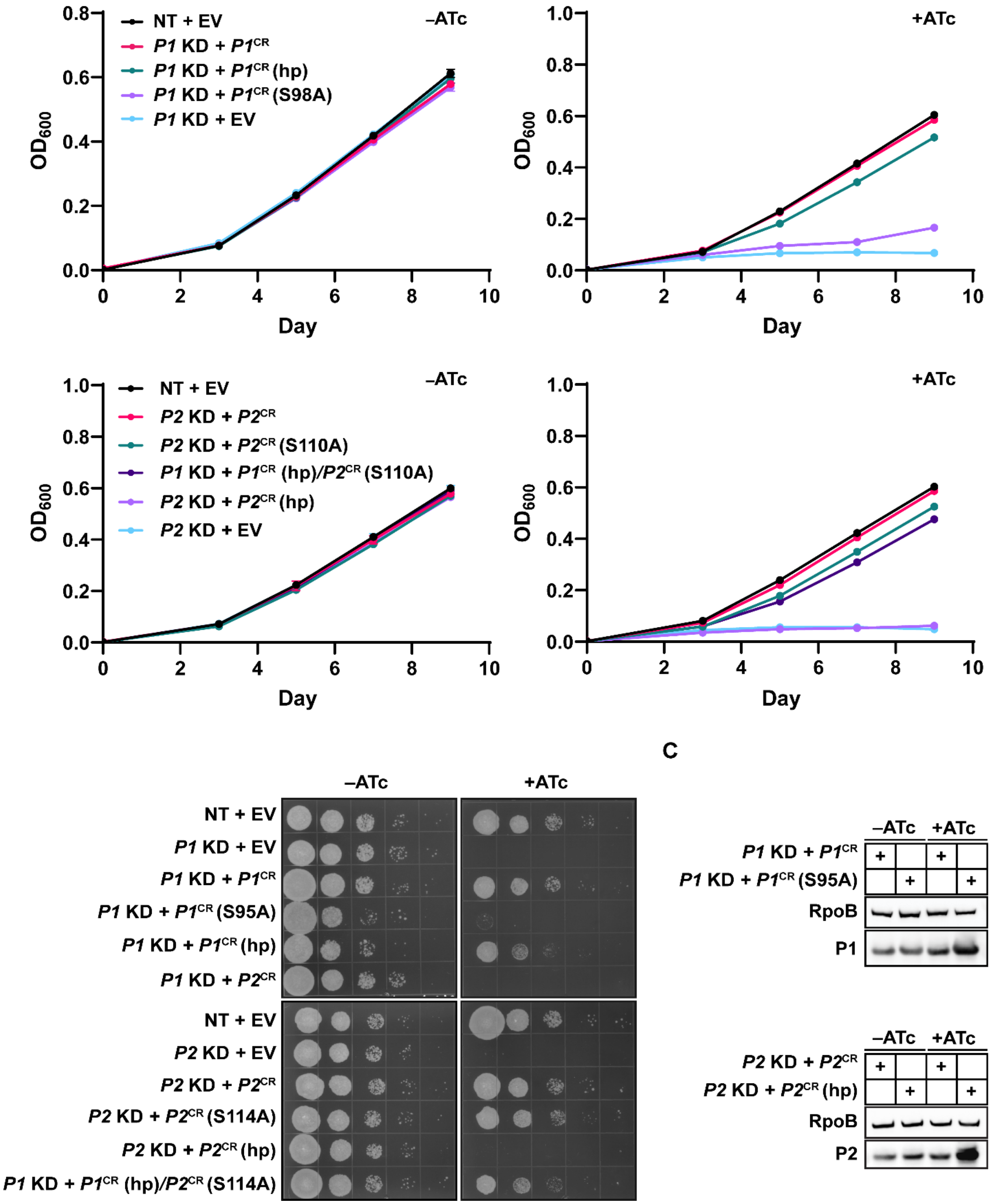

Supplemental Figure 1. The Mtb and Msmeg Clp complexes are functionally asymmetric *in vivo*.

(A) Liquid culture growth curves of the indicated Mtb CRISPRi strains in the absence or presence of the CRISPRi-inducer, anhydrotetracycline (ATc). NT = non-targeting sgRNA; EV = empty complementation vector; KD = knock-down; S98 = Mtb ClpP1 catalytic serine; CR = CRISPRi-resistant allele; S110 = Mtb ClpP2 catalytic serine; hp = hydrophobic patch mutations: P1 = S61A, Y63V, L83A, Y91V, P2 = Y75V, Y95V.

(B) Growth of the indicated Msmeg CRISPRi strains. S95 = Msmeg ClpP1 catalytic serine; S114 = Msmeg ClpP2 catalytic serine; hp = hydrophobic patch mutations: P1 = H58A, Y60V, L80A, Y88V, P2 = Y79V, Y99V.

(C) Immunoblot of CRISPRi-resistant Msmeg 3xFLAG-ClpP1 and 3xFLAG-ClpP1 (S95A) or Msmeg 3xFLAG-ClpP2 and 3xFLAG-ClpP2 (hp) mutant allele expressing strains. All strains also encode a *clpP1* (*P1* KD) or *clpP2* (*P2* KD) targeting sgRNA that silences the endogenous target gene in the presence of ATc.

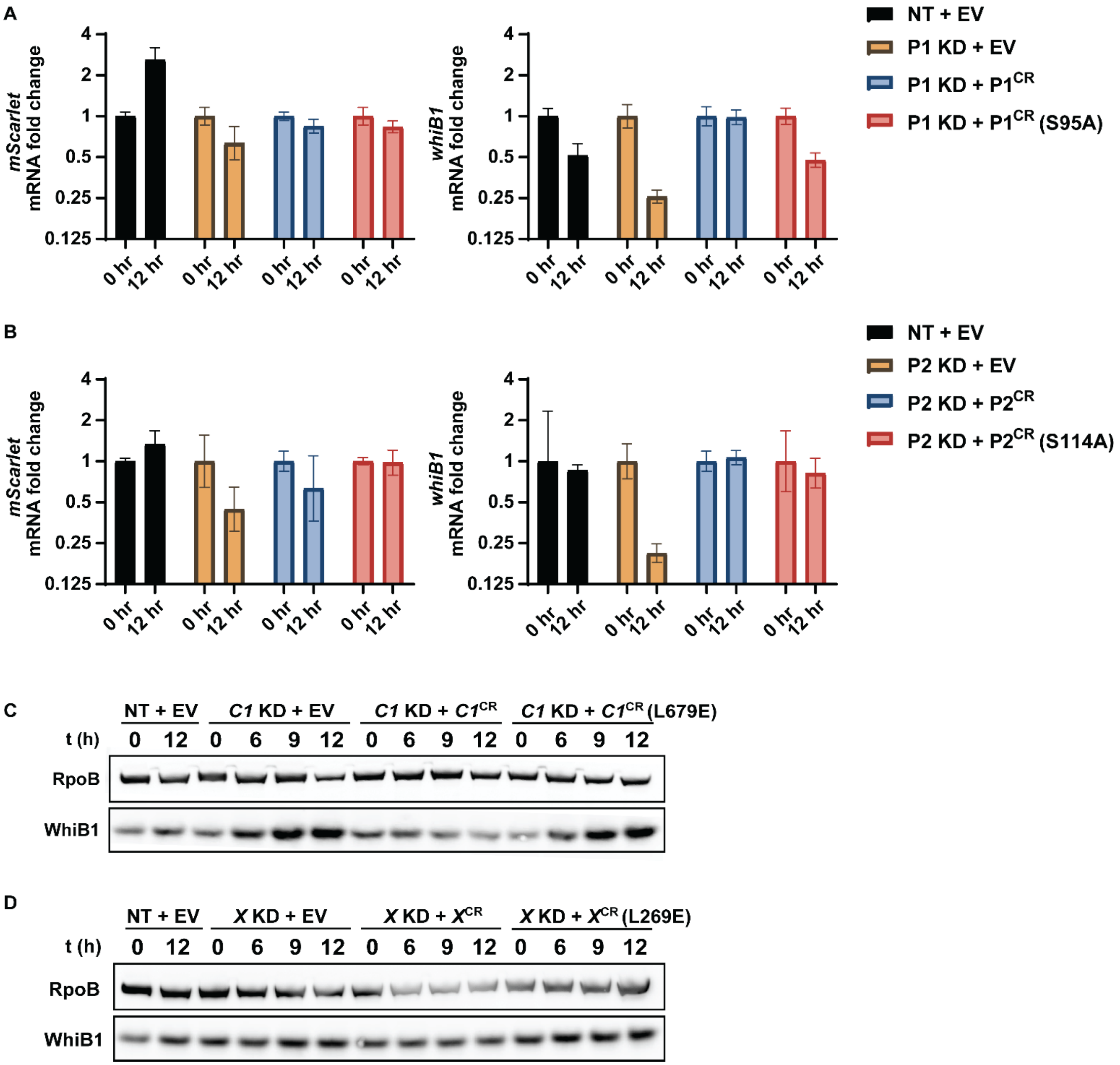

Supplemental Figure 2. Post-transcriptional regulation of mScarlet-YALAA and WhiB1

(A) *whiB1* and *mScarlet-YALAA* mRNA levels in Msmeg *clpP1* KD CRISPRi strains shown in **Figure 2B,E**. NT = non-targeting sgRNA; EV = empty complementation vector; KD = knock-down; S95 = Msmeg ClpP1 catalytic serine; CR = CRISPRi-resistant allele.

(B) *whiB1* and *mScarlet-YALAA* mRNA levels in Msmeg *clpP2* KD CRISPRi strains shown in **Figure 2B,E**. NT = non-targeting sgRNA; EV = empty complementation vector; KD = knock-down; CR = CRISPRi-resistant allele; S114 = Msmeg ClpP2 catalytic serine.

(C) Immunoblot measuring 3xFLAG-WhiB1 accumulation in the indicated Msmeg *clpC1* CRISPRi strains. t (h) = hours after ATc addition. NT = non-targeting sgRNA; EV = empty complementation vector; KD = knock-down; CR = CRISPRi-resistant allele; L679E = mutation of the ClpC1 LGF motif required for docking to ClpP2.

(D) Immunoblot measuring 3xFLAG-WhiB1 accumulation in the indicated Msmeg *clpX* CRISPRi strains. L269E = mutation of the ClpX LGF motif required for docking to ClpP2.

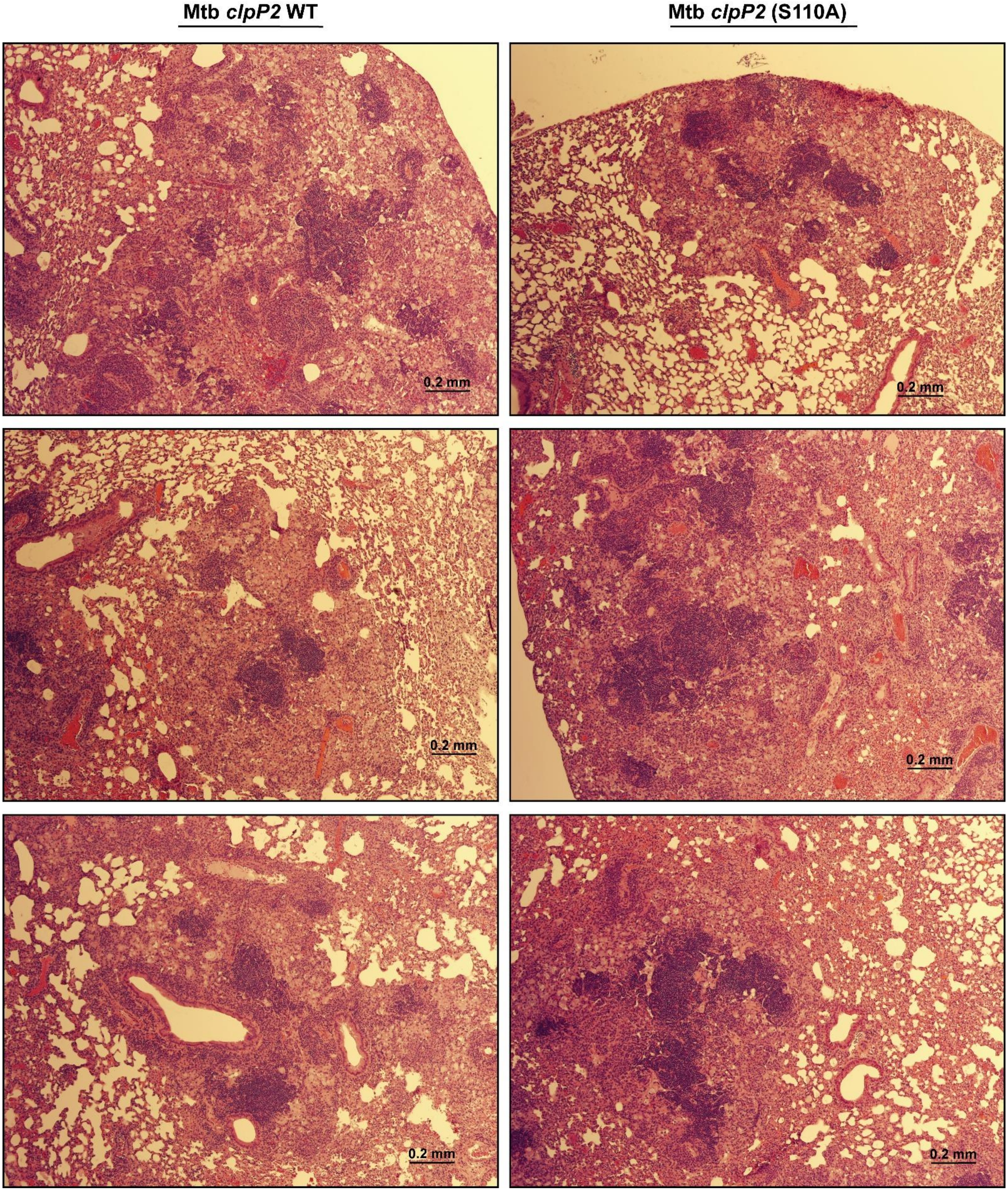

Supplemental Figure 3. Lung histopathology of mice infected with ClpP2 wild-type or ClpP2 S110A proteolytically-dead Mtb strains.
Lung sections were stained with hematoxylin and eosin after 136 days of infection with the indicated Mtb strains. Images are from three representative mice per strain. 4X magnification.

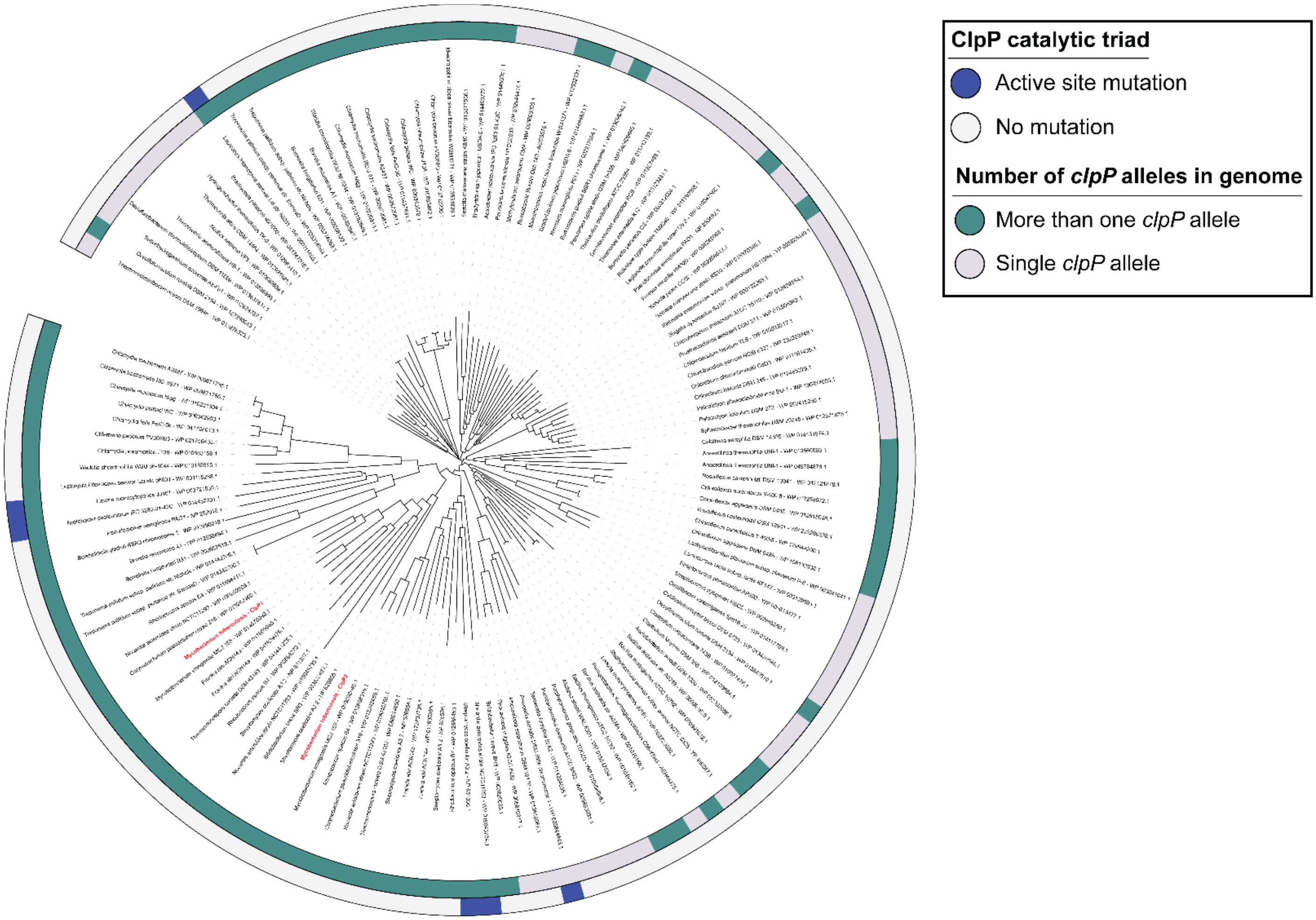

Supplemental Figure 4. Phylogenetic analysis of ClpP homologues reveals widespread but incomplete conservation of catalytic triad residues across different bacterial species.
Bacterial phylogenetic tree inferred from alignment of ClpP protein sequences across diverse bacterial species. Mtb ClpP1 and Mtb ClpP2 are labelled in red. Outer ring colors indicate the presence (“Active site mutation”) or absence (“No mutation”) of ClpP catalytic triad mutations. Inner ring colors indicate the presence of a single ("Single *clpP* allele”) or multiple ("More than one *clpP* allele”) *clpP* alleles in the indicated strain from that bacterial species.

Supplemental Videos
Time-lapse microscopy measuring mScarlet or mScarlet-YALAA accumulation in the indicated Msmeg CRISPRi strains. NT = non-targeting sgRNA; EV = empty complementation vector; KD = knock-down; S95 = Msmeg ClpP1 catalytic serine; CR = CRISPRi-resistant allele; S114 = Msmeg ClpP2 catalytic serine.

Supplemental Video 1 (SV1): NT + EV + mScarlet

Supplemental Video 2 (SV2): NT + EV + mScarlet-YALAA

Supplemental Video 3 (SV3): P1 KD + EV + mScarlet-YALAA

Supplemental Video 4 (SV4): P1 KD + $P1^{CR}$ + mScarlet-YALAA

Supplemental Video 5 (SV5): P1 KD + $P1^{CR}$ (S95A) + mScarlet-YALAA

Supplemental Video 6 (SV6): P2 KD + EV + mScarlet-YALAA

Supplemental Video 7 (SV7): P2 KD + $P2^{CR}$ + mScarlet-YALAA

Supplemental Video 8 (SV8): P2 KD + $P2^{CR}$ (S114A) + mScarlet-YALAA

Supplemental Data

Data S1: Alignment file for fig. S4

Data S2: Combined FASTA file for fig. S4

Data S3: Phylogenetic tree file for fig. S4

Supplemental Table 1: List of plasmids and oligonucleotides used in this work

Plasmids used in this work

Fig. used	Plasmid Name	Plasmid genotype	Plasmid description	Plasmid Map	Resistance marker
1A, S1A	pIRL58	Ptet(gB73)-Sth1 dCas9 Ptet(gB52)-Sth1 sgRNA P(gB37)-TetRCO(tetON) L5 attP only::Kan	Sth1 dCas9 CRISPRi plasmid optimized for use in *M. tuberculosis*. Sth1 dCas9 and the sgRNA are induced in the presence of ATc. This plasmid lacks the full L5 integrase and must be co-transformed pIRL19. Used as “non-targeting control,” NT.	https://benchling.com/s/seq-Gv9EfPn1vM7OMu6HPyU3	Kanamycin
1A, 1C, 2A, 2B, 2D, 2E, S1A, S2, SV1-8	pIRL19	Pmop-L5 Int AmpR (suicide plasmid)	The L5 phage Int protein is expressed from the mycobacterial optimized promoter (MOP). This backbone is non-replicating and non-integrating in mycobacteria.	https://benchling.com/s/seq-BAF0JelwBQN6D2dw5alf	Carbenicillin
1C, 2A, 2B, 2D, 2E, S1B, S2, S2, SV1-8	pIRL61	Ptet(gB74)-Sth1 dCas9 Ptet(gB52)-Sth1 sgRNA P(gB37)-TetRCO(tetON) L5 attP only::Kan (empty vector)	Sth1 dCas9 CRISPRi plasmid optimized for use in *M. smegmatis*. Sth1 dCas9 and the sgRNA are induced in the presence of ATc. This plasmid lacks the full L5 integrase and must be co-transformed pIRL19. Used as “non-targeting control,” NT.	https://benchling.com/s/seq-Y2epObngtSCffoZGvH7R?m=slm-bNXj3fgiKHgYPWtIRAN1	Kanamycin
1, 2, S1, S2, SV1-8	pIRL91	Tweety-attP only::NatR	Tweety::Nat integration backbone. This plasmid lacks the full Tweety integrase and must be co-transformed pIRL62. Used as “empty vector,” EV.	https://benchling.com/s/seq-kKMLRAnQ6uUC10KjRouu?m=slm-CDOd2SUUR3Tnsk6qv2Du	Nourseothricin/ Carbenicillin
1, 2, S1, S2, SV1-8	pIRL62	pGA-OXP15-intTw	The Tweety phage Int protein is expressed from the UV15 promoter. This backbone is non-replicating and non-integrating in mycobacteria.	https://benchling.com/s/seq-LtN3e9WbixCmPd0VOCAa	Carbenicillin
2A, 2B, 2D, 2E, S2, SV1-8	pIRL125	Giles-attP only::ZeoR	Giles::Zeo integration backbone. This plasmid lacks the full Giles integrase and must be co-transformed pIRL40. Used as “empty vector,” EV.	https://benchling.com/s/seq-zuJvoOwucTtyz8I8zNXa	Zeocin
2A, 2B, 2D, 2E, S2, SV1-8	pIRL40	Puv15-Giles Int	The Giles phage Int protein is expressed from the UV15 promoter. This backbone is non-replicating and non-integrating in mycobacteria.	https://benchling.com/s/seq-2PVXvHq0CfNPVacwz1Ku	Carbenicillin
1A, S1A	pINP220	pIRL91-PclpP1P2-clpP1P2 WT Tweety attP::Nat	pIRL91 backbone expressing a CRISPRi resistant WT allele of Mtb *clpP1P2* under its endogenous promoter.	https://benchling.com/s/seq-9YourcvXalkPpBxyQYOG?m=slm-KTFv9LY4n8Tg39jaPI7F	Nourseothricin/ Carbenicillin
1A, S1A	pIFD12	pIRL91-PclpP1P2-clpP1(S98A)P2 WT Tweety attP::Nat	pIRL91 backbone expressing a CRISPRi resistant allele of Mtb *clpP1*(S98A)*P2* under its endogenous promoter.	https://benchling.com/s/seq-DetnDVdqquxmMdeb5lHS?m=slm-lfhW0FsqRgQ8U8H58d4V	Nourseothricin/ Carbenicillin
1A, S1A	pIFD15	pIRL91-PclpP1P2-clpP1(hp)P2 WT Tweety attP::Nat	pIRL91 backbone expressing a CRISPRi resistant allele of Mtb *clpP1*(S61A,Y63V,L83A,Y91V) *P2* under its endogenous promoter.	https://benchling.com/s/seq-im6fz1Am7oPf4METrwnz?m=slm-AFEu6hIEAxpSm1i76cCL	Nourseothricin/ Carbenicillin
1A, S1A	pINP221	pIRL91-PclpP1P2-clpP2 WT Tweety attP::Nat	pIRL91 backbone expressing a CRISPRi resistant WT allele of Mtb *clpP2* under its endogenous promoter.	https://benchling.com/s/seq-a94cvVVEHS2Pbr115c58?m=slm-2GtP7DR0QYPNZpwNuDjM	Nourseothricin/ Carbenicillin

1A, S1A	pIFD13	pIRL91-PclpP1P2-clpP2(S110A) Tweety attP::Nat	pIRL91 backbone expressing a CRISPRi resistant allele of Mtb *clpP2*(S110A) under its endogenous promoter.	https://benchling.com/s/seq-9bMh7yqBvquEbgRgQBfV?m=slm-WXXcszUJuMnpUxIvJvRo	Nourseothricin/ Carbenicillin
1A, S1A	pIFD14	pIRL91-PclpP1P2-clpP2(hp) Tweety attP::Nat	pIRL91 backbone expressing a CRISPRi resistant allele of Mtb *clpP2*(Y75V,Y95V) under its endogenous promoter.	https://benchling.com/s/seq-LMATzb2mg10IZPaQV7vq?m=slm-0ig7I7eV5s67hCrAXT3m	Nourseothricin/ Carbenicillin
1A, S1A	pIFD16	pIRL91-PclpP1P2-clpP1(hp)P2(S110A) Tweety attP::Nat	"forced asymmetry strain": pIRL91 backbone expressing a CRISPRi resistant allele of Mtb *clpP1*(S61A,Y63V,L83A,Y91V) *P2*(S110A) under its endogenous promoter.	https://benchling.com/s/seq-8MT2HCGttz5MTZAkStGo?m=slm-wIh8CddbO6a8TJZlhKHT	Nourseothricin/ Carbenicillin
1C, 2A, 2B, 2E, S1B, S2 SV4	pINP175	pIRL91-PclpP1P2-clpP1P2 WT Tweety attP::Nat	pIRL91 backbone expressing a CRISPRi resistant WT allele of Msmeg clpP1P2 under its endogenous promoter.	https://benchling.com/s/seq-tSeImo1uUtvHKkRnY1ik?m=slm-F40bhHvJFI6YeYqW8zOI	Nourseothricin/ Carbenicillin
2A, 2B, 2E, S1B, S2, SV5	pINP233	pIRL91-PclpP1P2-clpP1(S95A)P2 WT Tweety attP::Nat	pIRL91 backbone expressing a CRISPRi resistant allele of Msmeg *clpP1*(S95A)*P2* under its endogenous promoter.	https://benchling.com/s/seq-zyttSTcVqCEVRy7LUVi5?m=slm-3eRaCFBh7Rlfp86e0Vqg	Nourseothricin/ Carbenicillin
S1B	pINP218	pIRL91-PclpP1P2-clpP1(hp)P2 WT Tweety attP::Nat	pIRL91 backbone expressing a CRISPRi resistant allele of Msmeg *clpP1*(H58A,Y60V,L80A,Y88V) *P2* under its endogenous promoter.	https://benchling.com/s/seq-eI6haKQNJ1e06R0f9qpR?m=slm-K56NSpNmSUZrfEj09kQv	Nourseothricin/ Carbenicillin
2A, 2B, 2E, S1B, S2, SV7	pINP173	pIRL91-PclpP1P2-clpP2 WT Tweety attP::Nat	pIRL91 backbone expressing a CRISPRi resistant WT allele of Msmeg clpP2 under its endogenous promoter.	https://benchling.com/s/seq-W99VXcz0YM3B0RqHHRkU?m=slm-T85jEbd9CF4j6g5hsEZj	Nourseothricin/ Carbenicillin
2A, 2B, 2E, S1B, S2, SV8	pINP234	pIRL91-PclpP1P2-clpP2(S114A) Tweety attP::Nat	pIRL91 backbone expressing a CRISPRi resistant allele of Msmeg *clpP2*(S114A) under its endogenous promoter.	https://benchling.com/s/seq-AcfMT0yxHs6cu6OsUCfh?m=slm-ogCiiYJVewL6ABSPkGq0	Nourseothricin/ Carbenicillin
S1B	pINP217	pIRL91-PclpP1P2-clpP2(hp) Tweety attP::Nat	pIRL91 backbone expressing a CRISPRi resistant allele of Msmeg *clpP2*(Y79V,Y99V) under its endogenous promoter.	https://benchling.com/s/seq-8Lyw93a3l8A528XR2IQt?m=slm-R8jbmKn14hw5d399wy37	Nourseothricin/ Carbenicillin
S1B	pINP237	pIRL91-PclpP1P2-clpP1(hp)P2(S114A) Tweety attP::Nat	"forced asymmetry strain": pIRL91 backbone expressing a CRISPRi resistant allele of Msmeg *clpP1*(H58A,Y60V,L80A,Y88V) *P2*(S114A) under its endogenous promoter.	https://benchling.com/s/seq-1zGt0h0eeuPnS0YIzCW2?m=slm-lZ5wpIONEX6MCbxUvito	Nourseothricin/ Carbenicillin
S1C	pIFD121	pIRL91-PclpP1P2-clpP1-3xFLAG,P2 WT Tweety attP::Nat	pIRL91 backbone expressing a CRISPRi resistant allele of Msmeg *clpP1*(C-terminal 3xFLAG),*P2* under its endogenous promoter.	https://benchling.com/s/seq-58h5Fb3j8R27Y1oFilOI?m=slm-q0K3pSR2pv008djqSBiU	Nourseothricin/ Carbenicillin
S1C	pIFD129	pIRL91-PclpP1P2-clpP1(S95A)-3xFLAG,P2 WT Tweety attP::Nat	pIRL91 backbone expressing a CRISPRi resistant allele of Msmeg *clpP1*(S95A, C-terminal 3xFLAG),*P2* under its endogenous promoter.	https://benchling.com/s/seq-cwqvzDRrCxcB6Tbosyye?m=slm-HxANRtktjjdfb5SOsPAx	Nourseothricin/ Carbenicillin
S1C	pIFD161	pIRL91-PclpP1P2- clpP2-3xFLAG Tweety attP::Nat	pIRL91 backbone expressing a CRISPRi resistant allele of Msmeg *clpP2*(C-terminal 3xFLAG) under its endogenous promoter.	https://benchling.com/s/seq-Icb6vCyTCjcPFvfLrZdb?m=slm-RvquItIWqZNdHs9eS07i	Nourseothricin/ Carbenicillin
S1C	pIFD157	pIRL91-PclpP1P2-clpP2(hp)-3xFLAG Tweety attP::Nat	pIRL91 backbone expressing a CRISPRi resistant allele of Msmeg *clpP2*(Y79V,Y99V, C-terminal 3xFLAG) under its endogenous promoter.	https://benchling.com/s/seq-wsmF38f18oH4Za0Ftv1C?m=slm-0v5uhQbiblb13TfOHfZw	Nourseothricin/ Carbenicillin
1B	pIFD116	pIRL91-PclpP1P2-clpP1P2 (processed) Tweety attP::Nat	pIRL91 backbone expressing a CRISPRi resistant allele of Msmeg *clpP1P2* (ΔM1-A16) under its endogenous promoter.	https://benchling.com/s/seq-sRlVLlNM984daltwA4hJ	Nourseothricin/ Carbenicillin
1B	pIFD117	pIRL91-PclpP1P2-clpP1(S95A)P2	pIRL91 backbone expressing a CRISPRi resistant allele of Msmeg *clpP1*(S95A)*P2* (ΔM1-	https://benchling.com/s/seq-apdgOK0tI61b1bTFfwRk?m=slm-RolFvG3SqmYmRp2DPGdm	Nourseothricin/ Carbenicillin

		(processed) Tweety attP::Nat	A16) under its endogenous promoter.		
1B	pIFD120	pIRL91-PclpP1P2-clpP1P2 (S114A, processed) Tweety attP::Nat	pIRL91 backbone expressing a CRISPRi resistant allele of Msmeg *clpP1P2* (ΔM1-A16, S114A) under its endogenous promoter.	https://benchling.com/s/seq-kZqp5h8V5hWsjesi9lWi	Nourseothricin/ Carbenicillin
2A, 2B, S2, SV2-8	pIFD24	pIRL125-P300-mScarlet-I-YALAA Giles attP::Zeo	pIRL125 backbone expressing *mScarlet-I*-YALAA driven by a constitutive p300 promoter.	https://benchling.com/s/seq-iEi9Ru4e102CID73Ixdp?m=slm-hEFpYOmvK5yQ41IEwTxP	Zeocin
SV1	pIFD23	pIRL125-P300-mScarlet-I Giles attP::Zeo	pIRL125 backbone expressing *mScarlet-I* driven by a constitutive p300 promoter.	https://benchling.com/s/seq-6pv223k7eij2HnUCT2zn?m=slm-g68UQABZGTfoEWcd3qMy	Zeocin
2E, S2	pIFD125	pIRL125-PclpP1P2-clpP1P2 WT Giles attP::Zeo	pIRL125 backbone expressing a CRISPRi resistant WT allele of Msmeg *clpP1P2* under its endogenous promoter.	https://benchling.com/s/seq-p9zAXxqHdUah7N5tnCZ0?m=slm-9lsCAdEW090oOSYGuHak	Zeocin
2E, S2	pIFD126	pIRL125-PclpP1P2-clpP1(S95A)P2 WT Giles attP::Zeo	pIRL125 backbone expressing a CRISPRi resistant allele of Msmeg *clpP1*(S95A)*P2* under its endogenous promoter.	https://benchling.com/s/seq-vmArtXoXpKmcPvxQ1Rzz?m=slm-ZsJFsNZCrglO19fosnUy	Zeocin
2E, S2	pIFD127	pIRL125-PclpP1P2-clpP2 WT Giles attP::Zeo	pIRL125 backbone expressing a CRISPRi resistant WT allele of Msmeg *clpP2* under its endogenous promoter.	https://benchling.com/s/seq-dCWNdYKzo3Rd9Xr5915x	Zeocin
2E, S2	pIFD128	pIRL125-PclpP1P2-clpP2(S114A) Giles attP::Zeo	pIRL125 backbone expressing a CRISPRi resistant allele of Msmeg *clpP2*(S114A) under its endogenous promoter.	https://benchling.com/s/seq-G6gbNMMfn7VWo1MMZUBb?m=slm-rRUpj0EuhmhqjAbvuAy5	Zeocin
2C, 2E, S2	pIRL35	pIRL91-PwhiB1-3xFLAG-whiB1 Tweety attP::Nat	pIRL91 backbone expressing a CRISPRi resistant allele of Msmeg *whiB1* with an N-terminal 3x-FLAG tag under its endogenous promoter.	https://benchling.com/s/seq-1THfeBfDZraikA28xAiF	Nourseothricin/ Carbenicillin
2C	pIRF49	pIRL91-PwhiB1-3xFLAG-whiB1(ΔK72-V84) Tweety attP::Nat	pIRL91 backbone expressing a CRISPRi resistant allele of Msmeg *whiB1*(ΔK72-V84) with an N-terminal 3x-FLAG tag under its endogenous promoter.	https://benchling.com/s/seq-2Nr2HqnyIfQH4OhHhDxa	Nourseothricin/ Carbenicillin
2D	pIRF8	pIRL91-PwhiB1-whiB1 Tweety attP::Nat	pIRL91 backbone expressing a CRISPRi resistant allele of Msmeg *whiB1* under its endogenous promoter.	https://benchling.com/s/seq-4D7PjqWgpn1RWVazJvwH	Nourseothricin/ Carbenicillin
2D	pIRF17	pIRL91-PwhiB1-whiB1(ΔK72-V84) Tweety attP::Nat	pIRL91 backbone expressing a CRISPRi resistant allele of Msmeg *whiB1*(ΔK72-V84) under its endogenous promoter.	https://benchling.com/s/seq-g4GoNnOqyAa7d7XkaerR	Nourseothricin/ Carbenicillin
3	pIRL4	Ptet-recT(gp61) Ptb21-T10M sacB oriM::Kan	The *recT* annealase from the Che9c mycobacteriophage is expressed from an ATc-regulated promoter. Used for single-stranded DNA recombineering with oIFD030 and oIFD054.	https://benchling.com/s/seq-6aMaBQNbeM95fKaOTHKY	Kanamycin
S2	pIFD124	pIRL125-PclpC1-clpC1 WT Giles attP::Zeo	pIRL125 backbone expressing a WT CRISPRi resistant allele of Msmeg *clpC1* under its endogenous promoter.	https://benchling.com/s/seq-RZ3xXIATz53V5KydlWUh	Zeocin
S2	pIFD132	pIRL125-PclpC1-clpC1 (L679E) WT Giles attP::Zeo	pIRL125 backbone expressing a CRISPRi resistant allele of Msmeg *clpC1* (L679) under its endogenous promoter.	https://benchling.com/s/seq-o27YSSUuAIkCkkNzpYmU	Zeocin
S2	pIFD123	pIRL125-PclpX-clpX WT Giles attP::Zeo	pIRL125 backbone expressing a WT CRISPRi resistant allele of Msmeg *clpX* under its endogenous promoter.	https://benchling.com/s/seq-JRgh904eFeO8LIqAYWcO	Zeocin
S2	pIFD136	pIRL125-PclpX-clpX (L269E) Giles attP::Zeo	pIRL125 backbone expressing a CRISPRi resistant allele of Msmeg *clpX* (L269) under its endogenous promoter.	https://benchling.com/s/seq-5rV5r9tXDv121TyovP1q	Zeocin

sgRNAs used in this work

Fig. used	sgRNA ID	Gene targeted	Gene name	sgRNA targeting sequence (5'–3')
1, 2, S1, S2, SV1	Non-targeting control	NA	NA	GGAGACGATTAATGCGTCTCG
1C, 2A, 2B, 2E, S1B, S2, SV1	MSMEG4673:clpP_Uncertain_ATGTCGTCGTCCACTTGGGAACC_23mer_v4PAMscore6	*ms4673*	*clpP1*	ATGTCGTCGTCCACTTGGGAACC
2A, 2B, 2E, S1B, S2, SV1	MSMEG4672:clpP_Uncertain_GTCCGCGGCCTGGATCTCCAGATC_24mer_v3PAMscore7	*ms4672*	*clpP2*	GTCCGCGGCCTGGATCTCCAGATC
1A, S1A	RVBD2461c:clpP1_Essential_GCTGGCGTCTTCGGCGGCCAGCA_23mer_v3PAMscore3	*rv2461c*	*clpP1*	GCTGGCGTCTTCGGCGGCCAGCA
1A, S1A	RVBD2460c:clpP2_Essential_GGCGGCCTGGATCTCCAGATC_21mer_v3PAMscore7	*rv2460c*	*clpP2*	GGCGGCCTGGATCTCCAGATC
2A	MSMEG4222:ftsZ_Uncertain_GCGCCGCTTGCCCTCGAA_18mer_v4PAMscore7	*ms4222*	*ftsZ*	GCGCCGCTTGCCCTCGAA
2D	MSMEG1919:MSMEGEG1919_Uncertain_GGCCCACTGTTTCCCACCGG_20mer_v3PAMscore7	*ms1919*	*whiB1*	GGCCCACTGTTTCCCACCGG
S2	MSMEGEG6091:MSMEGEG6091_Uncertain_ATGATCTCGTCCTGCGTCAGCTG_23mer_v3PAMscore6	*ms6091*	*clpC1*	ATGATCTCGTCCTGCGTCAGCTG
S2	MSMEG4671:clpX_Uncertain_ACCAGCGCGTTCTTGGGCTCGG_22mer_v3PAMscore8	*ms4671*	*clpX*	ACCAGCGCGTTCTTGGGCTCGG

qPCR primers used in this work

Fig. used	Target gene	Gene name	Forward primer (5'-3')	Reverse primer (5'-3')
S2	*ms1919*	*whiB1*	TTGCCCAGATCGCTGAC	CTGGCCCGACTCCAAAG
S2	*mScarlet-I*	*mScarlet-I*	AGGACGGTACCCTCATCTATAA	CCAACCCATCGTCTTCTTCT

Other oligonucleotides used in this work

Fig. used	Sequence (5'-3')	Notes
3	gcgggcaaccttccgaagcgccgagttcggcttcCtcggagtggtggtgtacacgcgggtgcatacacc	Mtb *rpsL* (K43R) SNP ssDNA recombineering oligomer (Mtb streptomycin resistance)
3	cgatatccagacggtgtgtctgggccaggcAgcAGcTgcggctgcggtgctgctggccgccggaacaccg	Mtb *clpP2* (S110A) ssDNA recombineering oligomer (coding strand)
3	ttctcagatccagccccaggcgcgc	Mtb *clpP2* F-amplification primer
3	gagcttccggtactcgagcaccg	Mtb *clpP2* R-amplification primer
3	cactccagcttcggggtcaaggag	Mtb *clpP2* Sanger sequencing primer
